# Supplementary material for: Temporal transcriptional regulation of mitochondrial morphology primes activity-dependent circuit connectivity
Source: Nat Commun. 2025 Sep 1;16:8173. doi: 10.1038/s41467-025-62908-2 (PMC12402207; doi:10.1038/s41467-025-62908-2)
Supplement: Supplementary file 1 — Supplementary Information File [file 41467_2025_62908_MOESM1_ESM.pdf]

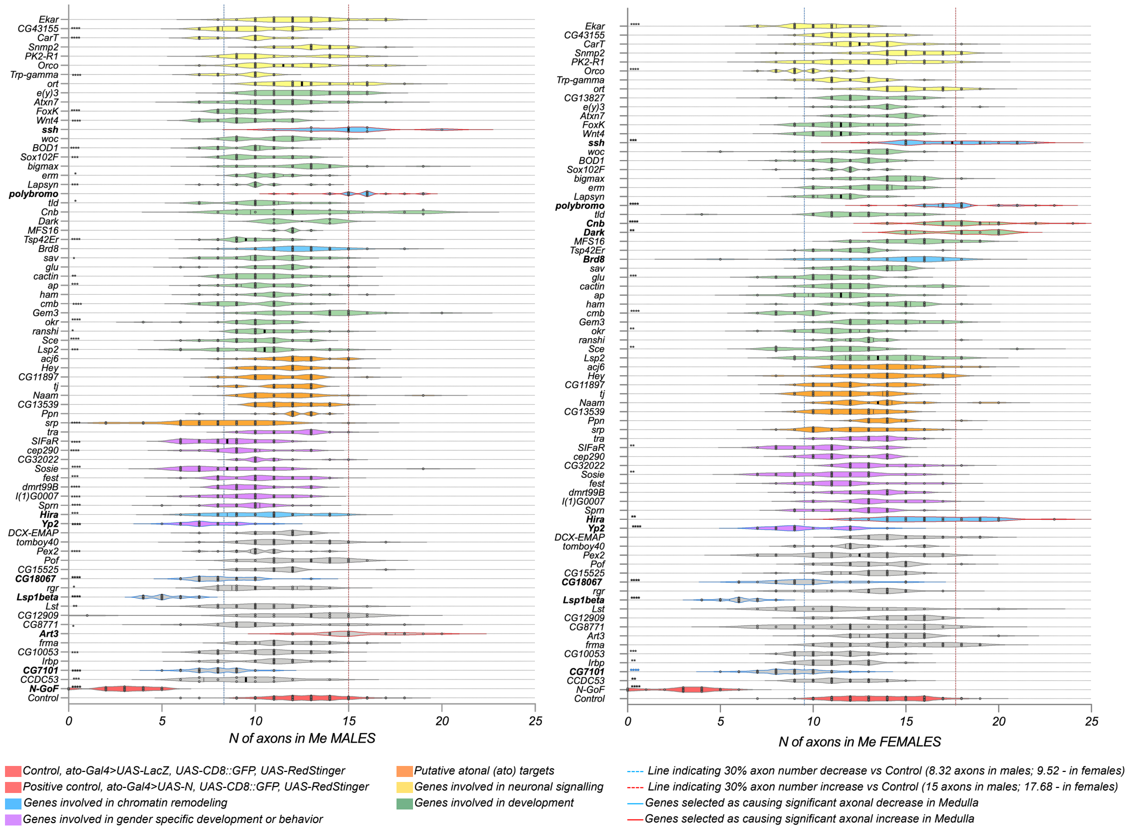

Supplementary Figure S1. RNAi screen of 74 genes expressed in DCNs

As a readout for the screen, a number of DCNs axons in the Medulla was used and compared to Control flies (*w^{1118};UAS-LacZ/+;ato-Gal4^{14A},UAS-CD8::GFP,UAS-RedStinger*). Flies with *Notch* overexpression were used as positive Control, (*N-GoF*) as it was shown earlier that Notch overexpression is preventing DCNs to establish their connections in Medulla[1]. We considered observed phenotypes as positive if changes in medulla innervation were equal to or greater than 30% of the mean axon number in Control flies (blue scattered line corresponds to a 30% decrease, and red – 30% increase values). Candidate genes (in bold) that were selected during the screen have blue (for decreased innervation) and red (for increased innervation) line borders on the plot. We divided genes by groups, according to known annotations of their function (corresponding colors with description in the figure legend). Crosses were set in the groups of five RNAi lines plus Control with each group. Adult flies were dissected at 3-7 days old. The final number of analyzed optic lobes in Control flies, 158 males & 154 females. In tested genes in the screen, *n* value varies from 8 to 38 in males and from 12 to 45 in females (depending on genotype and the number of repeats). Statistical analysis was done with GraphPad Prism 8 using Ordinary *One-way ANOVA* with Bonferroni's corrections for multiple comparisons. Adjusted P value, * *p<0.05*, ** *p≤0.01*, *** *p≤0.001*, **** *p≤0.0001*, *ns* – not significant.

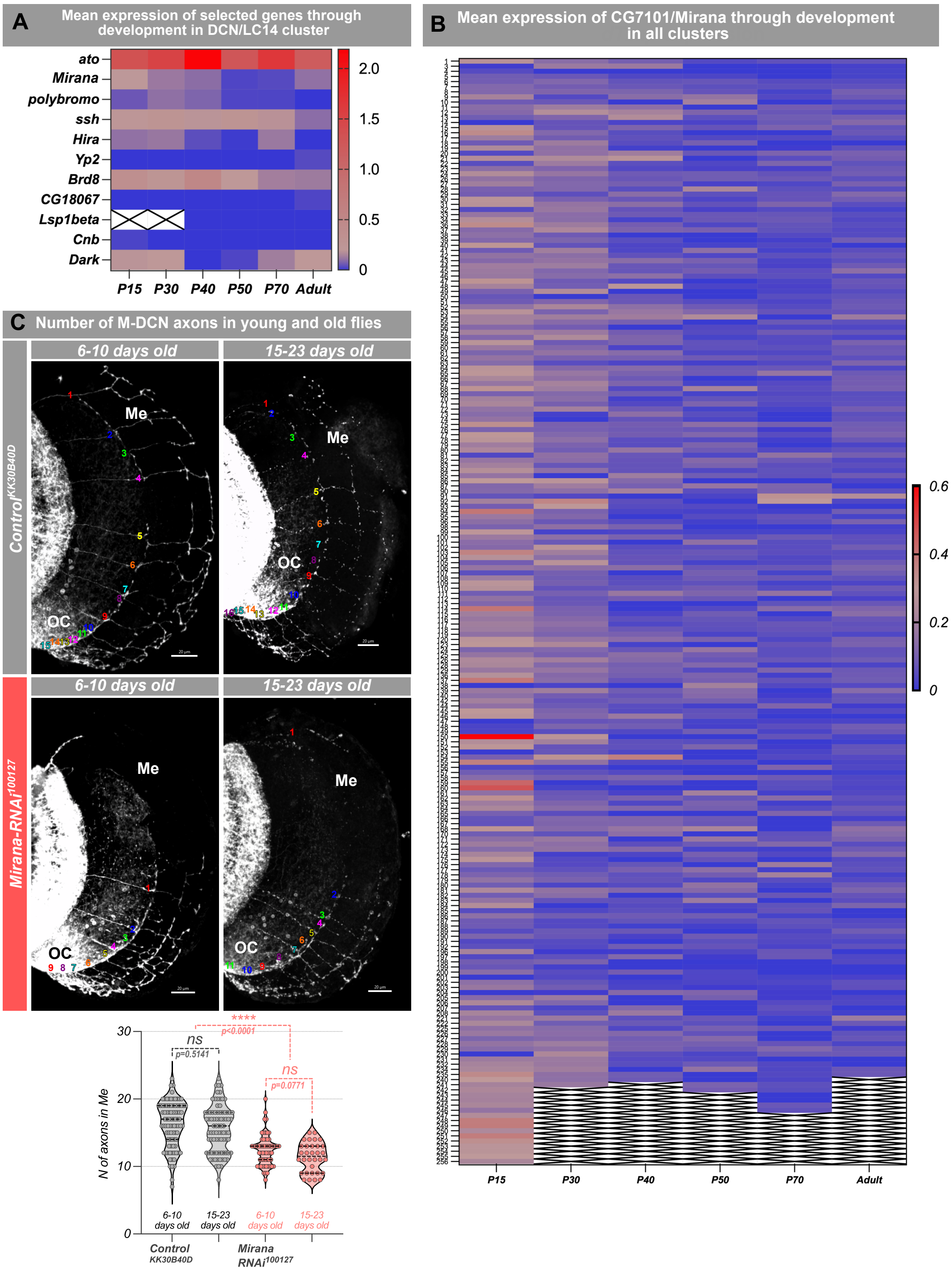

Supplementary Figure S2. scRNAseq analysis of a dataset from Ozel et al.,2020 [2] and ***medulla DCN axon analysis in young vs old individuals.***

Heat maps were generated from the available dataset of single-cell RNAseq of the fly visual system at different developmental stages. A – normalized mean expression of 10 candidate genes, with a significant change in the number of axons reaching Medulla, in DCN cluster at different developmental stages. B - normalized mean expression of *Mirana* throughout development in different neuronal clusters. C – Number of M-DCNs axons in young (6-10 days old) and old (15-23 days old) flies. OC – optic chiasm, Me – medulla, scale bar – 20µm. Statistical analysis was performed with GraphPad Prism 8 using the nonparametric Mann-Whitney U test. Adjusted P values, * $p<0.05$, ** $p\leq0.01$, *** $p\leq0.001$, **** $p\leq0.0001$, *ns* – not significant.

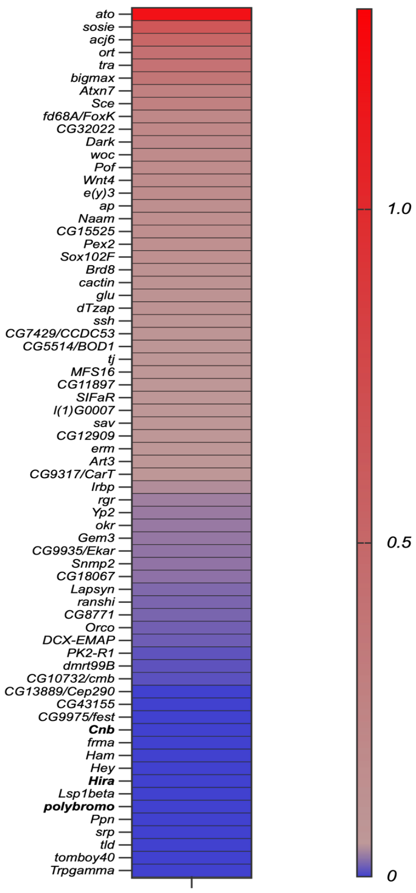

Gene	Adult_cluster 27 average expression	Gene	Adult_cluster 27 average expression
ato	1,203275	*erm*	0,055759
sosie	0,720877	*Art3*	0,051147
acj6	0,573652	*CG9317/CarT*	0,051073
ort	0,478456	*Irbp*	0,040991
tra	0,468061	*rgr*	0,031073
bigmax	0,43101	*Yp2*	0,028382
Atxn7	0,311296	*okr*	0,027964
Sce	0,308021	*Gem3*	0,027194
fd68A/FoxK	0,246225	*CG9935/Ekar*	0,025371
CG32022	0,235872	*Snmp2*	0,024921
Dark	0,212891	*CG18067*	0,023586
woc	0,2046	*Lapsyn*	0,018654
Pof	0,197071	*ranshi*	0,016617
Wnt4	0,187513	*CG8771*	0,016402
e(y)3	0,186956	*Orco*	0,014033
ap	0,166477	*DCX-EMAP*	0,012474
Naam	0,155915	*PK2-R1*	0,008305
CG15525	0,15306	*dmrt99B*	0,007954
Pex2	0,134948	*CG10732/cmb*	0,007155
Sox102F	0,132866	*CG13889/Cep290*	0
Brd8	0,124843	*CG43155*	0
cactin	0,106855	*CG9975/fest*	0
glu	0,10642	*Cnb*	0
dTzap	0,10325	*frma*	0
ssh	0,09377	*Ham*	0
CG7429/CCDC53	0,093226	*Hey*	0
CG5514/BOD1	0,078429	*Hira*	0
tj	0,076434	*Lsp1beta*	0
MFS16	0,066595	*polybromo*	0
CG11897	0,063581	*Ppn*	0
SIFaR	0,060799	*srp*	0
l(1)G0007	0,059513	*tld*	0
sav	0,056964	*tomboy40*	0
CG12909	0,056946	*Trpgamma*	0

Supplementary Figure S3. Upregulated genes in DCNs isolated clusters of our RNAseq experiment also could be found in dataset generated by Ozel et al.,2020 [19] in cluster 27_LC14, that correspond to DCNs.

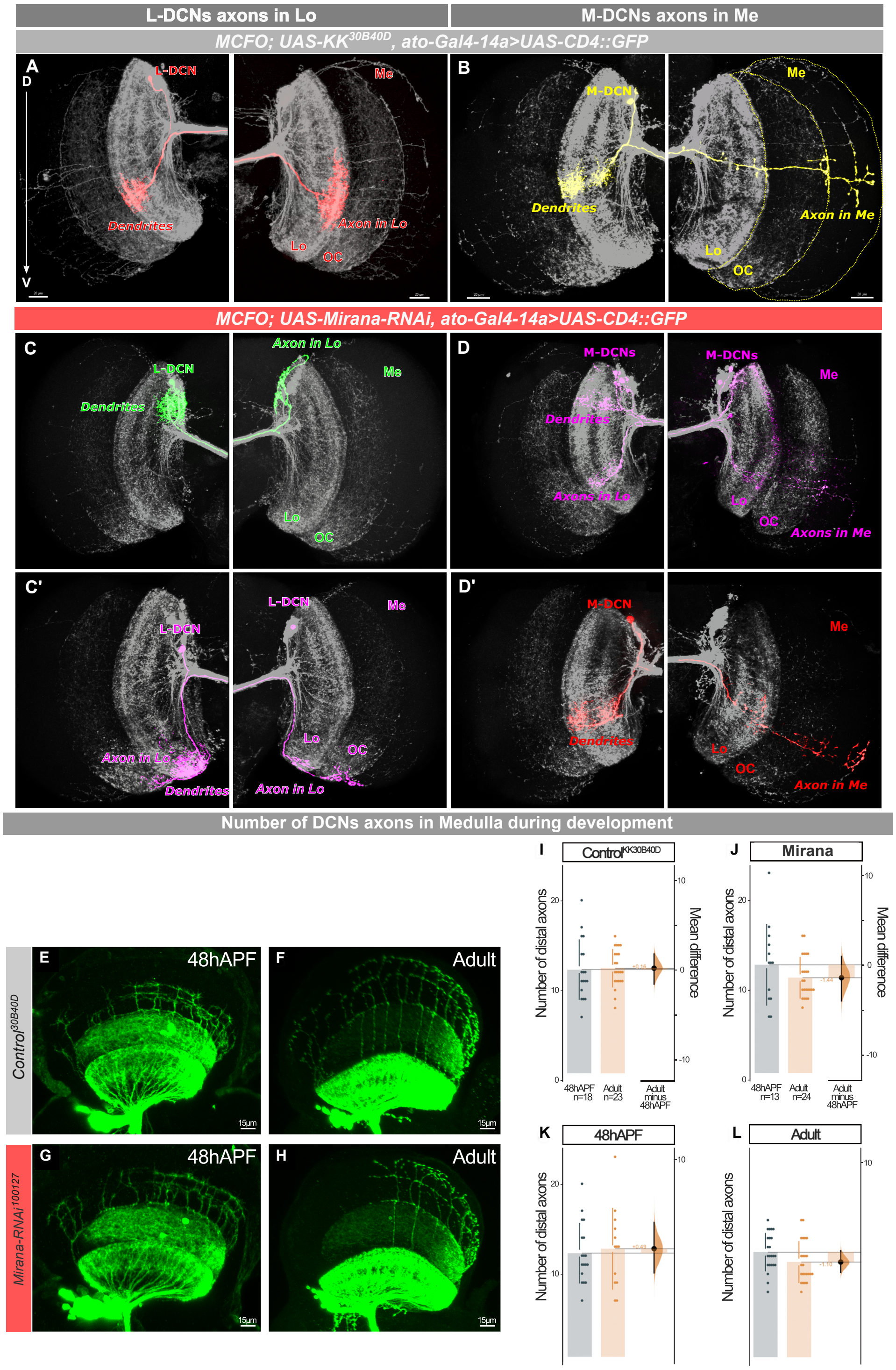

Supplementary Figure S4. Single cell labelling of DCN neurons using Multi Color Flip-out system.

A, B – L-DCNs (A) and M-DCNs (B) of control flies showing normal DCN morphology. C, C', D, D' – L-DCNs (C, C') and M-DCNs (D, D') of flies with *Mirana* depletion (*RNAi*^{100127}) showing no morphological defects on single cell level at adult stage. Lo – lobula., OC – optic chiasm, Me – medulla. Scale bar – 20μm. E – H – number of M-DCNs axons during development at P48 (E, G) and adult flies (F, G). E – F – Control flies (*w*^{1118}*; KK-UAS*^{30B40D}*; ato-Gal4-14a, UAS-CD8::GFP*). G – H – *Mirana*-RNAi flies (*w*^{1118}*; UAS-Mirana-RNAi*^{100127}*; ato-Gal4-14a,UAS-CD8::GFP*). I - L – quantification of medulla axons at two developmental points. Scale bar – 15μm.

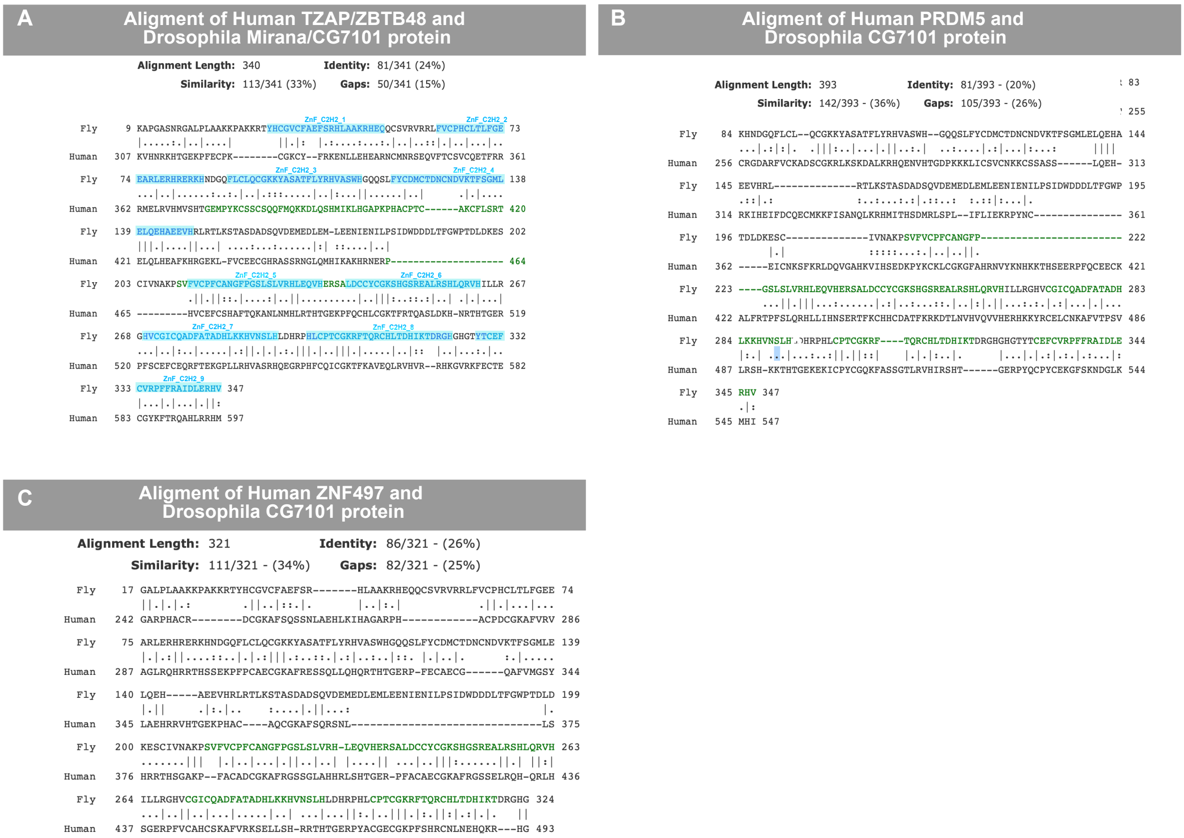

***Supplementary Figure S5.* Alignment of *Drosophila* CG7101 protein and human putative homologs**

A – Alignment of human TZAP/ZBTB48 and *Drosophila* CG7101 protein. ZnF_C2H2 domains of fly protein showed in blue. B - Alignment of human PRDM5 and *Drosophila* CG7101 protein. C - Alignment of human ZNF497 and *Drosophila* CG7101 protein. Alignments adopted from DIOPT, DRSC integrative ortholog prediction tool).

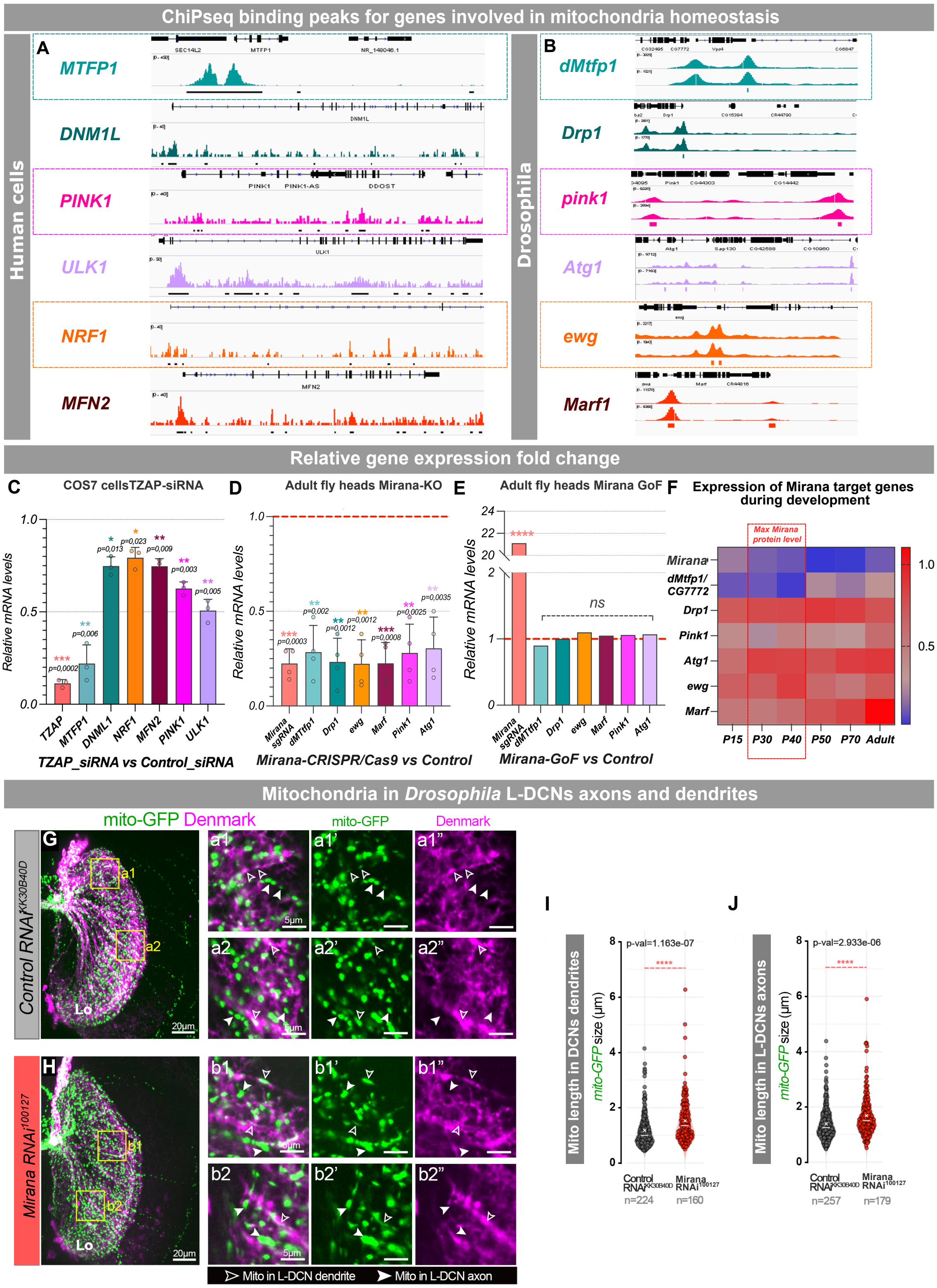

Supplementary Figure S6. Mirana is a conserved transcriptional factor that binds to a number of nuclear genes involved in mitochondria biology in flies and human cells

A, B – Analysis of available ChiPseq datasets from adult whole organism for fly Mirana (A) [25] and mammalian TZAP (B) [26] showing significant peaks at promoter regions of several nuclear genes involved in mitochondria biology. C - E – qRT-PCR measurement of relative mRNA levels of TZAP and Mirana target genes shows significant decrease in case of its downregulation with *Tzap_siRNA* in COS7 cells (C) and *nsyb-Gal4>Mirana_sgRNA;UAS-*

U^{M}-Cas9^{340007} in flies (D). Overexpression of *Mirana* (*nsyb-Gal4>UAS-Mirana^{F001205})* had no effect on downstream genes expression (E). F – temporal expression pattern in DCNs cluster of *Mirana* putative targets, involved in mitochondria biology (single cell RNAseq data from *Ozel et al.,2020* [19]). ΔCt values of the qRT-PCR experiment were compared using one-way ANOVA with Dunn correction for multiple comparisons. G - H – mitochondria labbeled with GFP in dendrites (labelled with DenMark-mCherry) of control (G, *w^{1118};UAS-myto-GFP/KK-UAS^{30B40D_60101}; ato-Gal4-14a, UAS-denmark.mCherry)* and *Mirana*-RNAi flies (H, w^{1118};UAS-myto-GFP/UAS-*Mirana*-RNAi^{100127};ato-Gal4-14a/UAS-denmark.mCherry). Arrowheads showing mitochondria in L-DCNs dendrites and axons. I - J – Quantification of GFP puncta length in L-DCNs dendrites (I) and axons (J). Statistical analyses were done using R software. Normal data distribution was first tested using the Shapiro-Wilk normality test. non-parametric tests were further performed: a two-sided Mann–Whitney U-test or a rank-based non-parametric Kruskal–Wallis test followed by a Dunn test when comparing more than two conditions. * $p<0.05$, ** $p\leq0.01$, *** $p\leq0.001$, **** $p\leq0.0001$, *ns* – not significant.

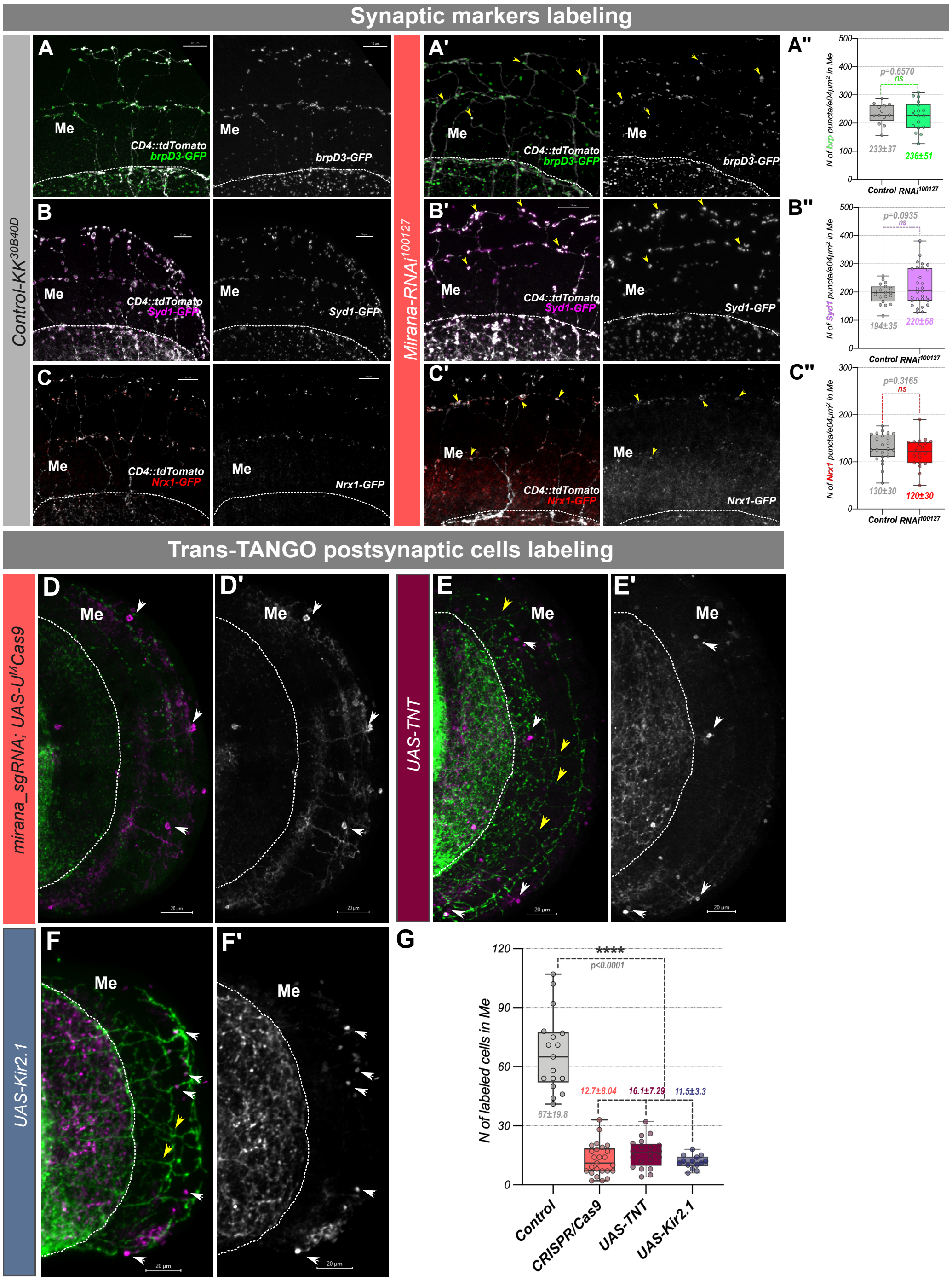

Supplementary Figure S7. Synaptic marker and transTango labeling of DCNs postsynaptic connectivity

A - C'' – labeling of synaptic markers in M-DCNs axons in *Mirana-RNAi*^{100127} background showed no significant difference to controls (*w*^{1118}*; UAS-KK*^{40D30B}), scale bar 10µm. BrpD3, Syd1, and Nrx1 were present at the presynaptic sites in quantities comparable to control condition (A", B", C"). GFP positive puncta were automatically counted using IMARIS software in medulla, and normalized to corresponding medulla area. Puncta counting: BrpD3 – 13 optic lobes (control) and 18 optic lobes (*RNAi*^{100127}), Syd1 – n=19 optic lobes (control) and 29 optic lobes (*RNAi*^{100127}), Nrx1 – n=24 optic lobes in (control) and 21(*RNAi*^{100127}). D - F' – TransTango anterograde postsynaptic labeling of DCNs connectivity in case of *Mirana* downregulation using CRISPR/Cas9 approach (*UAS-myrGFP.QUAS-mtdTomato-3xHA;transTANGO/UAS-Mirana_sgRNA;ato-Gal4*^{14a}*/UAS-U*^{M}*Cas9*^{340007}) (D - D'), and DCNs silencing with Tetanus toxin (E, E', n=20 optic lobes) and potassium channel overexpression with *UAS-Kir2.1* (F, F', n=14 optic lobes). DCNs

expressing *myrGFP* on the cellular membrane are shown in green (D, E, F) and postsynaptic labeled cells in magenta (merged picture) and grayscale (D', E', F'). G - Postsynaptic connectivity was analyzed by manually counting cell bodies in Medulla (white arrowheads) labeled with dsRed fluorescence (magenta, merged picture – D, E, F, and grayscale – D', E', F'), including all cells with weak or strong labeling to reveal all potential connections. M-DCNs axons visible in medulla (yellow arrowheads) show drastically reduced connectivity with neighboring cells. Scale bar 20μm. Statistical analysis was done using an unpaired Student's t-test with Welch's correction for axon counting, and one-way ANOVA with Bonferroni corrections for multiple comparisons in TransTango labeling analysis. * $p<0.05$, ** $p\leq0.01$, *** $p\leq0.001$, **** $p\leq0.0001$, *ns* – not significant.

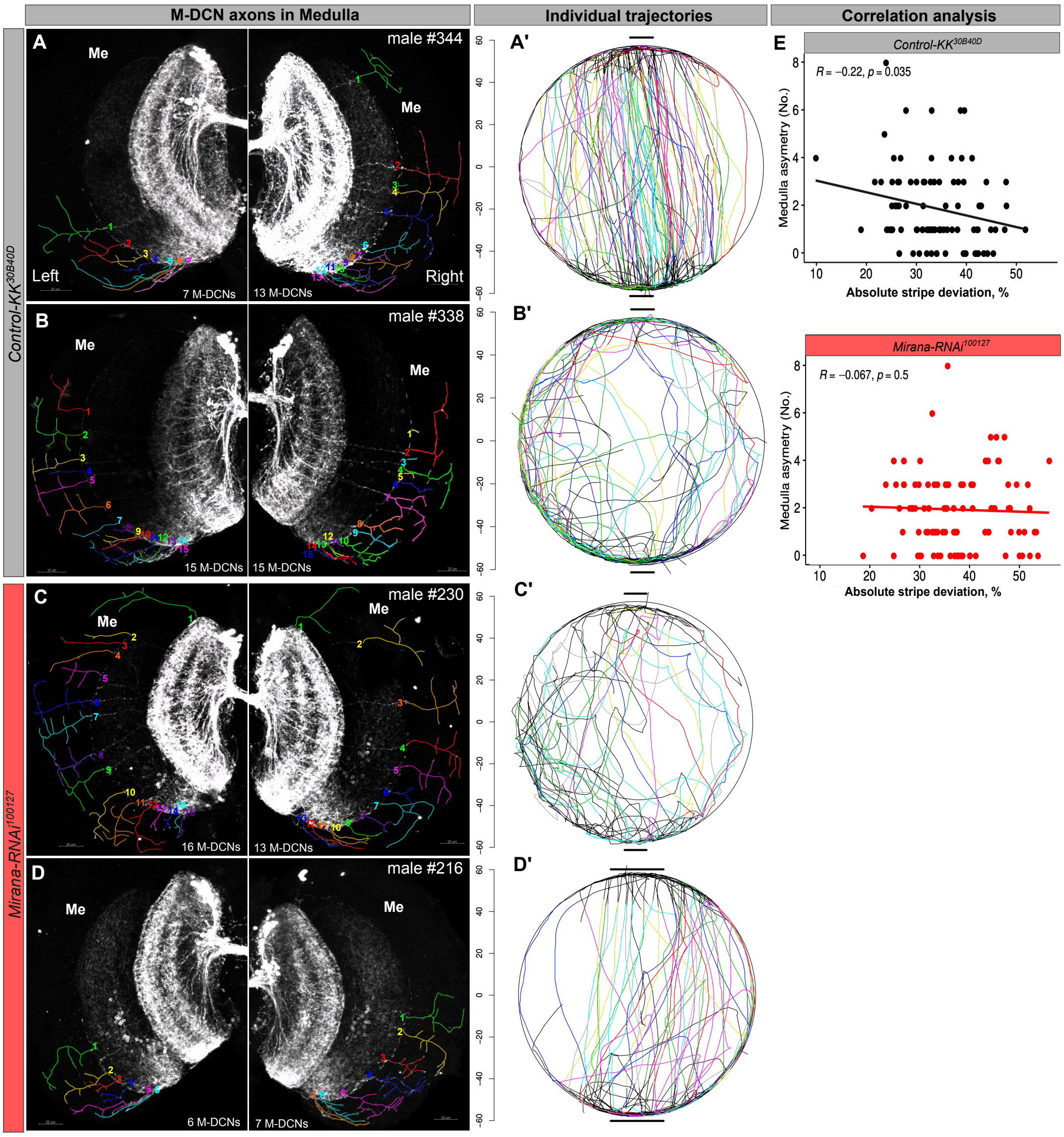

Supplementary Figure S8. Correlation analysis of Individual trajectories in Buridan assay and DCN circuit anatomy

A, D – DCN neurons in individual control males (#344 and #338, *W+;UAS-KK^{40D30B}; ato-Gal4^{14A},UAS-CD4::GFP)* and males with *Mirana* downregulation (#230 and #216, *W+;UAS-Mirana-RNAi^{100127};ato-Gal4^{14A},UAS-CD4::GFP)* showing the different degrees of asymmetry*.* A' r- D' – corresponding individual behavioral paths in the Buridan assay. E – correlation analysis of individual DCN innervation asymmetry in Medulla and absolute stripe deviation (125 control flies and 135 *RNAi^{100127}* flies. Both, males and females in equal ratio). Me – medulla, Lo – lobula, OC – optic chiasm. Statistical analysis was done using one-way ANOVA with the Kruskal-Wallis test for the behavioral experiment, correlation analysis was performed using Spearman rank correlation.

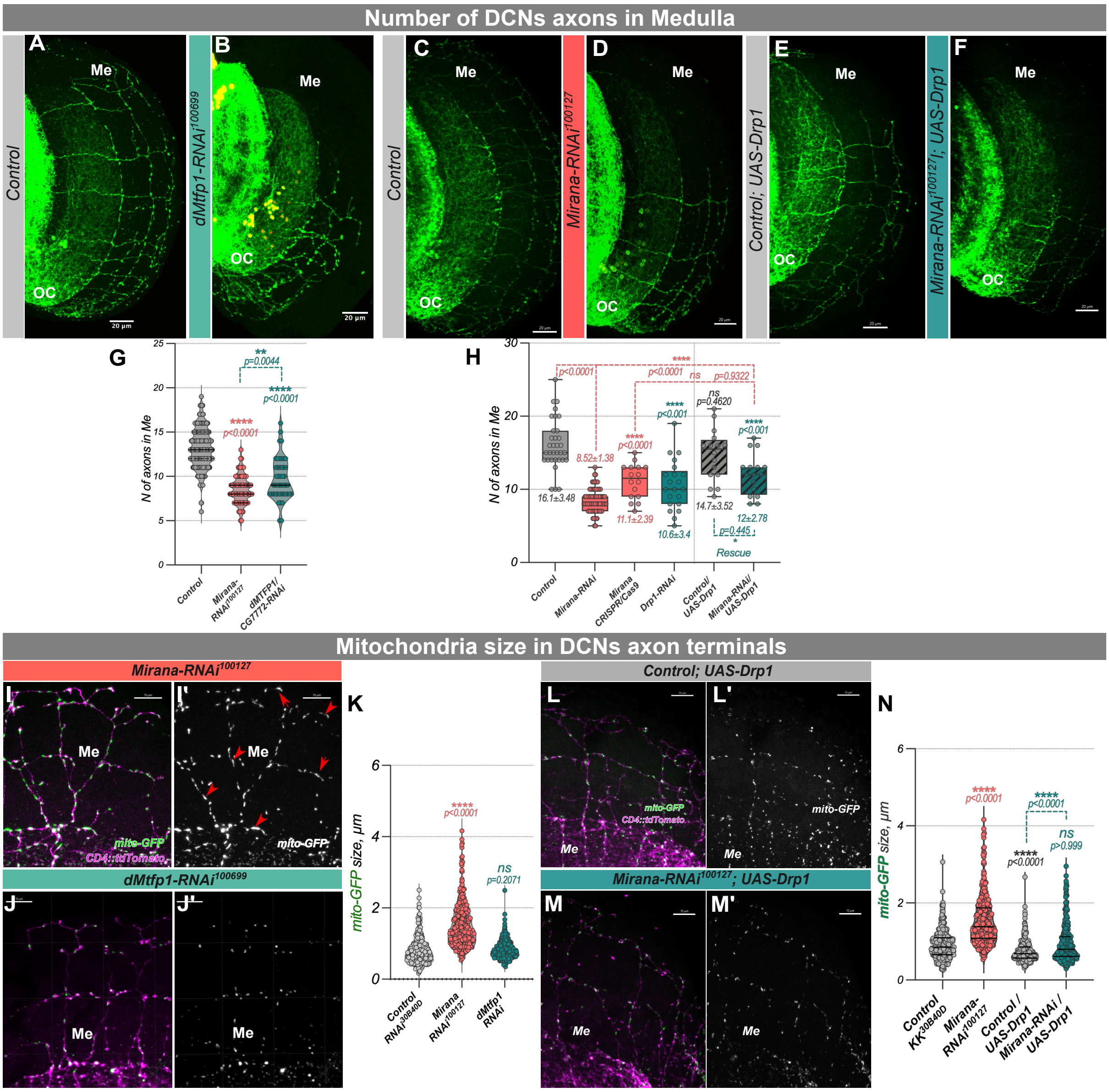

Supplementary Figure S9. Downregulation of Mirana target genes involved in fission partially phenocopies Mirana depletion phenotype. A - H – number of M-DCNs axons. Constitutive downregulation of *CG7772/dMtfp1* (A, B, G) and *Drp1* (H) decreases number of axons innervating medulla. However, overexpression of *Drp1* in *Mirana* knockdown background (L - N) does not rescue normal medulla innervation (F, H). I - N – mitochondria size in medulla axons of DCNs with red arrowheads pointing on elongated mitochondria in *Mirana* knockdown background. Downregulation of *CG7772/dMtfp1* (J - J', K) or *Drp1* (Figure 6, E) was not causing morphological changes in mitochondria. Overexpression of Drp1 in *Mirana* knockdown background was able to rescue mitochondrial elongation phenotype (M – N). Mitochondrial size was manually measured through the Z-stack of a confocal image of one/two axons in the Optic chiasm and Medulla region in at least 3/4 different brains of each sex. Scale bar – 10µm. Statistical analysis was done using the nonparametric Kruskal-Wallis test with Dunn correction for multiple comparisons and one-way ANOVA with Bonferroni correction for multiple comparisons respectively. ** p<0.05, ** p≤0.01, *** p≤0.001, **** p≤0.0001, ns* – not significant.

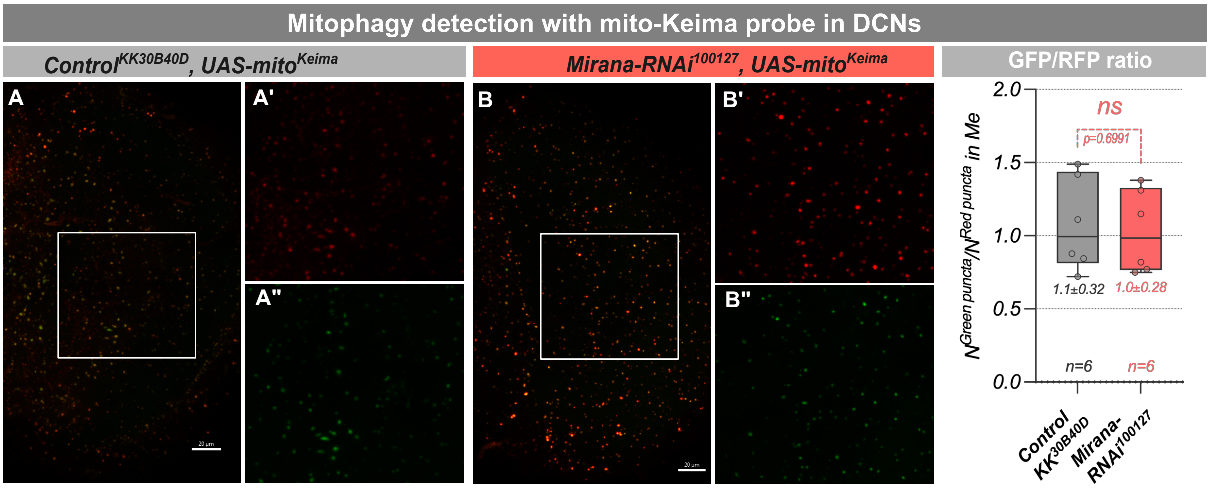

Supplementary Figure S10. Mithophagy detection using mito-Keima probe in DCNs. Confocal imaging of flies expressing transgenic mito-Keima sensor. At the pH of the normal mitochondria matrix (pH 8.0), mito-Keima exhibits an excitation peak at 440 nm, and when delivered to the lysosome, the excitation peak shifts to 586 nm. This large shift allows sensitive detection of the mitophagy process, and in our study revealed no difference in mitophagy levels between Mirana depleted and Control DCNs in adult animals. DCNs were imaged in not fixed brains, right after dissection in green and red channel. Then number of green and red puncta were analyzed using IMARIS software, and calculated GFP/RFP ratios as described before [46].

Supplementary Table 2. Number of normalized peaks for CG7101-GFP ChiP-Seq data sets from whole female fly (data generated by ModEnCode project)

Peaks found

Category	Gene name Drosophila	Human homolog	Function	Number of peaks by gene
	CG7101	***TZAP***	Transcription factor	1
Mito transport	*milt*	*TRAK1/TRAK2*	Mitochondria transport	6
Mito transport	*Miro*	*RHOT1/RHOT2*	Mitochondria transport	1
Mitochondria dynamic and architecture	***Drp1***	***DRP1***	Mitochondrial fision	1
Mitochondria dynamic and architecture	***ewg***	***NRF1***	Mitochondria biogenesis	2
Mitochondria dynamic and architecture	***Marf***	***MFN1/2***	Mitochondrial fusion	1
Mitochondria dynamic and architecture	*Mitofilin*	*IMMT/MIC60*	Cristae architecture	2
	cnc/Nrf2	*NFE2L1/NFE2L2/NFE2L3*	oxidative stress genes regulation	6
	Tom20	*MTOMM20*	Mitochondrial import	1
Autophagy and quality control	***park***	***PRKN***	Quality control	1
Autophagy and quality control	***Pink1***	***PINK1***	Quality control	1
Autophagy and quality control	*ref(2)P*	*SQSTM1*	Quality control	1
Autophagy and quality control	***Atg1***	***ULK1***	Mitophagy	2
Autophagy and quality control	*Atg12*	*ATG12*	Autophagy	3
Autophagy and quality control	*Atg5*	*ATG5*	Autophagic vesicle formation	2
Autophagy and quality control	*Atg7*	*ATG7*	Autophagy	2
mtDNA replication and maintenance	*bor*	*ATAD3A*	Component of nucleoids	2
mtDNA replication and maintenance	*SCSα1*	*SUCLG1*	Mitochondrial nucleotide synthesis	1
mtDNA replication and maintenance	*mtDNA-helicase*	*TWNK*	mtDNA replication	1
mtDNA replication and maintenance	*mtSSB*	*SSBP1*	mtDNA replication	1
mtDNA replication and maintenance	*mtTFB1*	*TFB1M*	Mitochondrial transcription	1
mtDNA replication and maintenance	*mtTFB2*	*TFB2M*	Mitochondrial transcription	2

Category	Gene name Drosophila	Human homolog	Function	Number of peaks by gene
Mitochondria translation	*mRpL1*	*MRPL1*	Mitoribosomes subunits	1
Mitochondria translation	*mRpL10*	*MRPL10*	Mitoribosomes subunits	1
Mitochondria translation	*mRpL11*	*MRPL11*	Mitoribosomes subunits	1
Mitochondria translation	*mRpL13*	*MRPL13*	Mitoribosomes subunits	1
Mitochondria translation	*mRpL16*	*MRPL16*	Mitoribosomes subunits	1
Mitochondria translation	*mRpL17*	*MRPL17*	Mitoribosomes subunits	1
Mitochondria translation	*mRpL18*	*MRPL18*	Mitoribosomes subunits	2
Mitochondria translation	*mRpL19*	*MRPL19*	Mitoribosomes subunits	1
Mitochondria translation	*mRpL2*	*MRPL2*	Mitoribosomes subunits	1
Mitochondria translation	*mRpL20*	*MRPL20*	Mitoribosomes subunits	1
Mitochondria translation	*mRpL21*	*MRPL21*	Mitoribosomes subunits	1
Mitochondria translation	*mRpL23*	*MRPL23*	Mitoribosomes subunits	2
Mitochondria translation	*mRpL24*	*MRPL24*	Mitoribosomes subunits	1
Mitochondria translation	*mRpL27*	*MRPL27*	Mitoribosomes subunits	3
Mitochondria translation	*mRpL28*	*MRPL28*	Mitoribosomes subunits	1
Mitochondria translation	*mRpL32*	*MRPL32*	Mitoribosomes subunits	1
Mitochondria translation	*mRpL33*	*MRPL33*	Mitoribosomes subunits	1
Mitochondria translation	*mRpL34*	*MRPL34*	Mitoribosomes subunits	1
Mitochondria translation	*mRpL35*	*MRPL35*	Mitoribosomes subunits	1
Mitochondria translation	*mRpL37*	*MRPL37*	Mitoribosomes subunits	1
Mitochondria translation	*mRpL39*	*MRPL39*	Mitoribosomes subunits	1
Mitochondria translation	*mRpL4*	*MRPL4*	Mitoribosomes subunits	1
Mitochondria translation	*mRpL41*	*MRPL41*	Mitoribosomes subunits	1
Mitochondria translation	*mRpL43*	*MRPL43*	Mitoribosomes subunits	1
Mitochondria translation	*mRpL44*	*MRPL44*	Mitoribosomes subunits	1
Mitochondria translation	*mRpL47*	*MRPL47*	Mitoribosomes subunits	1
Mitochondria translation	*mRpL48*	*MRPL48*	Mitoribosomes subunits	1
Mitochondria translation	*mRpL50*	*MRPL50*	Mitoribosomes subunits	1
Mitochondria translation	*mRpL51*	*MRPL51*	Mitoribosomes subunits	1
Mitochondria translation	*mRpL52*	*MRPL52*	Mitoribosomes subunits	2
Mitochondria translation	*mRpL53*	*MRPL53*	Mitoribosomes subunits	1
Mitochondria translation	*mRpL54*	*MRPL54*	Mitoribosomes subunits	1
Mitochondria translation	*mRpL55*	*MRPL55*	Mitoribosomes subunits	2
Mitochondria translation	*mRpL9*	*MRPL9*	Mitoribosomes subunits	1

Category	Gene name Drosophila	Human homolog	Function	Number of peaks by gene
Complex I	*ND-18*	*NDUFS4*	Supernumerary subunit	1
Complex I	*ND-20*	*NDUFS7*	Core subunit	1
Complex I	*ND-23*	*NDUFS8*	Core subunit	1
Complex I	*ND-39*	*NDUFA9*	Supernumerary subunit	2
Complex I	*ND-42*	*NDUFA10*	Supernumerary subunit	1
Complex I	*ND-51*	*NDUFV1*	Core subunit	1
Complex I	*CIA30*	*NDUFAF1*	Assembly factor	2
Complex II	*SdhA*	*SDHA*	Subunit	2
Complex II	*Sdhaf3*	*SDHAF3*	Assembly factor	1
Complex II	*SdhB*	*SDHB*	Subunit	1
Complex II	*SdhC*	*SDHC*	Subunit	2
Complex II	*Sirup*	*SDHAF4*	Assembly factor	1
Complex III	*Ttc19*	*TTC19*	Assembly factor	1
Complex III	*Ox*	*UQCR10*	Supernumerary subunit	2
Complex IV	*Ccdc56*	*COA3*	Assembly factor	1
Complex IV	*COX5A*	*COX5A*	Citochrome C oxidase subunit	1
Complex IV	*COX5B*	*COX5B*	Suplementary subunit	1
Complex IV	*COX7A*	*COX7A1*	Suplementary subunit	1
Complex V	*ATPsynB*	*ATP5PB*	Core subunit	1
Complex V	*ATPsynC*	*ATP5MC1/ATP5MC2/ATP5MC3*	Core subunit	3
Complex V	*ATPsynD*	*ATP5PD*	Core subunit	3
CoQ	*qless*	*PDSS1*	CoQ biosynthesis	2

No peaks

Gene name Drosophila	Human homolog	Function	Number of peaks by gene	Category
srl	***PCG1α/PPARGC1A***	Transcriptional coactivator	0	
opa1	***OPA1***	Mitochondrial fusion	0	Mitochondrial dynamics
Mic26-27	*MIC26-27*	Cristae architecture	0	Mitochondrial dynamics
Slc25A46a	***SLC25A46***	Mitochondria dynamics	0	Mitochondrial dynamics
Fis1	***FIS1***	Mitochondria fission	0	Mitochondrial dynamics
PolG1/tam	*POLG*	mtDNA replication	0	mtDNA replication and transcription
TFAM	*TFAM*	mtDNA transcription and replication	0	mtDNA replication and transcription
mTTF	*MTERF1*	mtDNA transcription	0	mtDNA replication and transcription
mRpL15	*MRPL15*	Mitoribosome large subunit	0	Mitochondrial translation
tko	*MRPS12*	Mitoribosome small subunit	0	Mitochondrial translation
mRpL41	*MRPL41*	Mitoribosome subunit	0	Mitochondrial translation
mt:ND2	*MT_ND2*	Core subunit	0	Complex I
sloth1	*SMIM4/UQCC5*	Assembly factor	0	Complex III
sloth2	*Brawnin/UQCC6*	Assembly factor	0	Complex III
Bcs1	*BCS1L*	Assembly factor	0	Complex III
mt:Coi	*MT-CO1*	Core subunit	0	Complex IV
Coa7	*COA7*	Assembly factor	0	Complex IV
Coa8	*COA8*	Assembly factor	0	Complex IV
Surf1	*SURF1*	Assembly factor	0	Complex IV
Scox	*SCO1/SCO2*	Assembly factor	0	Complex IV

Supplementary Table 2. Number of normalized peaks for CG7101-GFP ChipSeq data sets from whole female fly (ModENCODE project). We were able to detect putative binding of CG7101/Mirana protein to numerous genes involved in mitochondria function and homeostasis.
