# Supplementary material for: Temporal transcriptional regulation of mitochondrial morphology primes activity-dependent circuit connectivity
Source: Nat Commun. 2025 Sep 1;16:8173. doi: 10.1038/s41467-025-62908-2 (PMC12402207; doi:10.1038/s41467-025-62908-2)
Supplement: Supplementary file 2 — Reporting Summary [file 41467_2025_62908_MOESM2_ESM.pdf]

nature portfolio

Corresponding author(s): *Double-anonymous peer review submissions: write DAPR and your manuscript number here instead of author names.*

Last updated by author(s): *YYYY-MM-DD*

Reporting Summary

Nature Portfolio wishes to improve the reproducibility of the work that we publish. This form provides structure for consistency and transparency in reporting. For further information on Nature Portfolio policies, see our Editorial Policies and the Editorial Policy Checklist.

Statistics

For all statistical analyses, confirm that the following items are present in the figure legend, table legend, main text, or Methods section.

n/a	Confirmed	
☐	☒	The exact sample size (*n*) for each experimental group/condition, given as a discrete number and unit of measurement
☐	☒	A statement on whether measurements were taken from distinct samples or whether the same sample was measured repeatedly
☐	☒	The statistical test(s) used AND whether they are one- or two-sided<br>*Only common tests should be described solely by name; describe more complex techniques in the Methods section.*
☐	☒	A description of all covariates tested
☐	☒	A description of any assumptions or corrections, such as tests of normality and adjustment for multiple comparisons
☐	☒	A full description of the statistical parameters including central tendency (e.g. means) or other basic estimates (e.g. regression coefficient) AND variation (e.g. standard deviation) or associated estimates of uncertainty (e.g. confidence intervals)
☐	☒	For null hypothesis testing, the test statistic (e.g. *F*, *t*, *r*) with confidence intervals, effect sizes, degrees of freedom and *P* value noted<br>*Give P values as exact values whenever suitable.*
☒	☐	For Bayesian analysis, information on the choice of priors and Markov chain Monte Carlo settings
☒	☐	For hierarchical and complex designs, identification of the appropriate level for tests and full reporting of outcomes
☒	☐	Estimates of effect sizes (e.g. Cohen's *d*, Pearson's *r*), indicating how they were calculated

Our web collection on statistics for biologists contains articles on many of the points above.

Software and code

Policy information about availability of computer code

Data collection	no software was used in data collection
Data analysis	software used in data analysis is described in the methods section of the manuscript

For manuscripts utilizing custom algorithms or software that are central to the research but not yet described in published literature, software must be made available to editors and reviewers. We strongly encourage code deposition in a community repository (e.g. GitHub). See the Nature Portfolio guidelines for submitting code & software for further information.

Data

Policy information about availability of data

All manuscripts must include a data availability statement. This statement should provide the following information, where applicable:

- Accession codes, unique identifiers, or web links for publicly available datasets
- A description of any restrictions on data availability
- For clinical datasets or third party data, please ensure that the statement adheres to our policy

The Data required to support the finding of this paper are available in the manuscript and supplementary files. Raw data are available upon request.

Research involving human participants, their data, or biological material

Policy information about studies with human participants or human data. See also policy information about sex, gender (identity/presentation), and sexual orientation and race, ethnicity and racism.

Reporting on sex and gender	Not applicable
Reporting on race, ethnicity, or other socially relevant groupings	Not applicable
Population characteristics	Not applicable
Recruitment	Not applicable
Ethics oversight	Not applicable

Note that full information on the approval of the study protocol must also be provided in the manuscript.

Field-specific reporting

Please select the one below that is the best fit for your research. If you are not sure, read the appropriate sections before making your selection.

☒ Life sciences ☐ Behavioural & social sciences ☐ Ecological, evolutionary & environmental sciences

For a reference copy of the document with all sections, see nature.com/documents/nr-reporting-summary-flat.pdf

Life sciences study design

All studies must disclose on these points even when the disclosure is negative.

Sample size	Sample sizes were not predefined based on statistical methods but were chosen based on the standard established in the field as reported in previous publications.
Data exclusions	No data was excluded
Replication	Experiments were repeated at least two times from independent crosses. All replications attempts were successful.
Randomization	Individual flies were randomly selected from fly vials at required developmental stage and adult age.
Blinding	When experiments involved multiple conditions (at least two), quantifications were done without knowing the genotype.

Reporting for specific materials, systems and methods

We require information from authors about some types of materials, experimental systems and methods used in many studies. Here, indicate whether each material, system or method listed is relevant to your study. If you are not sure if a list item applies to your research, read the appropriate section before selecting a response.

Materials & experimental systems

n/a	Involved in the study	
☐	☒	Antibodies
☐	☒	Eukaryotic cell lines
☐	☐	Palaeontology and archaeology
☐	☒	Animals and other organisms
☐	☐	Clinical data
☐	☐	Dual use research of concern
☐	☐	Plants

Methods

n/a	Involved in the study	
☐	☒	ChIP-seq
☐	☐	Flow cytometry
☐	☐	MRI-based neuroimaging

Antibodies

Antibodies used	Rabbit anti-GFP 1:1000 Invitrogen A-11122<br>Mouse anti-GFP 1: Invitrogen A-11120<br>Rabbit anti-DsRed 1:300 Clonetech 632496

	Rabbit anti-HA 1:300 Cell signaling technologies 3724S<br>Rat anti-FLAG (DYKDDDDK) 1:200 Novus Biologicals NBP1-06712SS<br>Mouse anti-V5-Tag 1:500 BioRad MCA2894<br>Donkey anti-rabbit Alexa-488 Life ThermoFisher A21206<br>Goat anti-mouse Alexa-488 Life ThermoFisher A11029<br>Donkey anti-mouse Alexa-555 Life ThermoFisher A31572<br>Goat anti-mouse Alexa-555 Life ThermoFisher A21424
Validation	All used antibodies have been published previously, and are used to enhance of detect genomicaly tagged proteins.

Eukaryotic cell lines

Policy information about cell lines and Sex and Gender in Research

Cell line source(s)	COS7 cells
Authentication	Cell lines were not authenticated
Mycoplasma contamination	All cell lines tested negative for Mycoplasma. COS7 cells (ATCC) were grown in Dulbecco's modified Eagle medium (DMEM, Gibco) supplemented with 10% fetal bovine serum (FBS), 1% L-glutamine (Invitrogen), and 1% penicillin-streptomycin (Invitrogen) and tested monthly for mycoplasma contamination (MycoAlert Mycoplasma Detection Kit, Lonza). Cells were plated in 8-well glass bottom Ibidi (80827; Ibidi), at a density of 1.5 × 104 per well precoated with poly-L-lysine (1 mg/L, Sigma). The following day, cells were transfected with 0.1 µg/well of pcDNA3-HA, 0.1 µg/well of pCB6-HA-mitoGFP 65, and 10 pmol/well of TZAP stealth siRNA (s6568, ThermoFisher) or AllStars negative control (Qiagen 1027281), with Lipofectamine 2000 (0.5 µl/well, Invitrogen) in 50 µl of Opti-MEM I (Gibco), without medium change, according to the manufacturer's instructions.
Commonly misidentified lines<br>(See ICLAC register)	Not applicable

Palaeontology and Archaeology

Specimen provenance	Not applicable
Specimen deposition	Not applicable
Dating methods	Not applicable

☐ Tick this box to confirm that the raw and calibrated dates are available in the paper or in Supplementary Information.

Ethics oversight	Not applicable

Note that full information on the approval of the study protocol must also be provided in the manuscript.

Animals and other research organisms

Policy information about studies involving animals; ARRIVE guidelines recommended for reporting animal research, and Sex and Gender in Research

Laboratory animals	Wild type rats, Drosophila melanogaster<br>Fly strains<br>w1118; +/+; ato-Gal4-14a Hassan Lab A3<br>w1118; +/+; ato-Gal4-14a, UAS-CD8::GFP, UAS-RedStinger Hassan Lab GUAS 217<br>w1118; UAS-LacZ/+; ato-Gal4-14a Hassan Lab A18<br>w1118; ; ato-Gal4-14a, UAS-CD8::GFP Hassan Lab A31<br>w1118; ; ato-Gal4-14a, UAS-CD4::GFP Hassan Lab<br>w1118; Sp/CyO ; ato-Gal4-14a, UAS-CD4::tdTomato/TM3 Hassan Lab Gg84N<br>w1118; ; nsyb-Gal4 Hassan Lab Gg110N<br>P{ry[+t7.2]=hsFLP}1, y[1] w[*]; P{w[+mC]=UAS-N.intra.GS}2/CyO;MKRS/TM2 BDSC 52008<br>w1118; UAS-CCDC53-RNAiKK30B VDRC 110316<br>w1118; UAS-CG7101-RNAiKK30B40D VDRC 100127<br>w1118/UAS-Irbp-RNAiKK30B40D; ; VDRC 110409<br>w1118; UAS-CG10053-RNAiKK30B VDRC 110453<br>w1118; UAS-frma-RNAiKK30B VDRC 102309<br>w1118; UAS-Art3-RNAiKK30B40D VDRC 109448<br>w1118; UAS-CG8771-RNAiKK40D VDRC 102650<br>w1118; UAS-CG12909-RNAiKK30B VDRC 104356<br>w1118; UAS-Lst-RNAiKK40D VDRC 106861<br>w1118; ; UAS-Lsp1-beta-RNAiGD VDRC 35583<br>w1118; ; UAS-rgr-RNAiGD VDRC 6290

w1118; UAS-CG18067-RNAiGD VDRC 4726
w1118; UAS-CG15525-RNAiKK30B VDRC 109005
w1118; UAS-Pof-RNAiKK30B40D VDRC 100546
w1118; UAS-Pex2-RNAiKK30B VDRC 108578
w1118; UAS-tomboy40-RNAiKK VDRC 105557
w1118; UAS-DCX-EMAP-RNAiKK30B VDRC 106573
w1118; ; UAS-Yp2-RNAiGD VDRC 50157
w1118; UAS-Hira-RNAiKK30B VDRC 106989
w1118; UAS-Sprn-RNAiKK30B40D VDRC 107555
w1118; UAS-l(1)G0007-RNAiGD VDRC 31908
w1118; UAS-dmrt99B-RNAiKK30B VDRC 105019
w1118; UAS-fest-RNAiKK30B40D VDRC 106300
w1118; ; UAS-Sosie-RNAiGD VDRC 33806
w1118; UAS-CG32022-RNAiKK30B VDRC 100506
w1118; UAS-cep290-RNAiKK30B40D VDRC 106725
w1118; UAS-SIFaR-RNAiGD VDRC 1783
w1118; UAS-tra-RNAiGD VDRC 2560
w1118; UAS-srp-RNAiKK VDRC 109521
w1118; UAS-Ppn-RNAiKK VDRC 108005
w1118; UAS-CG13539-RNAiKK30B VDRC 108798
w1118; UAS-Naam-RNAiKK30B VDRC 102796
w1118; UAS-tj-RNAiKK30B40D VDRC 108255
w1118; UAS-CG11897-RNAiKK30B40D VDRC 105174
w1118; UAS-Hey-RNAiKK40D VDRC 103570
w1118; UAS-Lsp2-RNAiKK30B VDRC 109979
w1118; UAS-Sce-RNAiKK30B VDRC 106328
w1118; UAS-ranshi-RNAiKK30B VDRC 107393
w1118; UAS-okr-RNAiKK30B VDRC 104323
w1118/UAS-Gem3-RNAiGD; ; VDRC 49505
w1118; UAS-cmb-RNAiKK40D VDRC 109767
w1118; UAS-ham-RNAiGD VDRC 40763
w1118; UAS-ap-RNAiKK30B40D VDRC 101511
w1118; UAS-cactin-RNAiGD VDRC 32718
w1118; UAS-glu-RNAiGD VDRC 10937
w1118; UAS-sav-RNAiKK30B40D VDRC 101323
w1118; UAS-Brd8-RNAiGD VDRC 49989
w1118; UAS-Tsp4Er-RNAiKK30B VDRC 104950
w1118; UAS-MFS16-RNAiKK30B40D VDRC 108635
w1118; UAS-Dark-RNAiKK30B VDRC 104215
w1118; UAS-Cnb-RNAiKK30B VDRC 106213
w1118; UAS-tld-RNAiKK30B VDRC 100930
w1118; UAS-polybromo-RNAiKK30B VDRC 108618
w1118; UAS-Lapsyn-RNAiKK30B40D VDRC 102333
w1118; UAS-erm-RNAiKK40D VDRC 106313
w1118; UAS-bigmax-RNAiKK30B VDRC 110630
w1118; UAS-Sox102F-RNAiGD VDRC 19022
w1118; UAS-BOD1-RNAiKK40D VDRC 105166
w1118; UAS-woc-RNAiGD VDRC 20994
w1118; UAS-ssh-RNAiKK30B VDRC 107998
w1118; UAS-Wnt4-RNAiKK30B40D VDRC 104671
w1118; UAS-FoxK-RNAiKK30B VDRC 1010151
w1118; UAS-Atxn7-RNAiKK30B VDRC 102078
w1118; UAS-e(y)3-RNAiKK30B VDRC 105946
w1118; UAS-CG13827-RNAiKK30B VDRC 101466
w1118; UAS-ort-RNAiKK30B VDRC 107363
w1118; UAS-Trp-gamma-RNAiKK30B40D VDRC 105280
w1118; UAS-Orco-RNAiKK40D VDRC 100825
w1118; UAS-PK2-R1-RNAiKK30B VDRC 103822
w1118; UAS-Snmp2-RNAiKK30B40D VDRC 101136
w1118; UAS-CarT-RNAiKK30B VDRC 101145
w1118; UAS-CG43155-RNAiKK30B40D VDRC 101483
w1118; UAS-Ekar-RNAiKK30B VDRC 102351
w1118; UAS-CG7101-RNAiGD VDRC 27849
GD library control: w1118; ; VDRC 60000
KK library control: y, w1118; P{attP, y[+], w[3`] VDRC 60100
KK-30B40D control: y, w1118; 40D-UAS VDRC 60101
y-, w-; PBac{CG7101-GFP.FPTB}VK00033 / TM6C, Sb BDSC 67655
w1118; ; UAS-CG7101, 3xHA FlyORF F001205
P{ry[+t7.2]=hsFLP}1, y[1] w[1118]; P{y[+t7.7] w[+mC]=HD_CFD00394}attP40/CyO-GFP VDRC-HD-CFD 341305
P{ry[+t7.2]=hsFLP}12, y[1] w[*]; ;PBac{y[+]-attP-3B=UAS-uMCas9}VK00033 VDRC-HD-CFD 340007
P{ry[+t7.2]=hsFLP}12, y[1] w[*]; P{y[+t7.7] w[+mC]=UAS-uMCas9}attP40 VDRC-HD-CFD 340002
UAS-Nrx1::GFP / UAS-Nrx1::GFP (Y); GlaBc / CyO, 3xp3-GFP, w- Sigrist Lab, FU Berlin
1194
w[*]; P{w[+mC]=UAS-brpD3.GFP}TR725 BDSC 36292
w; UAS-syd1-GFP / CyO; Dr / TM6B Hiesinger Lab, Berlin
W1118; UAS-myto-GFP / CyO Hiesinger Lab, Berlin

w1118; UAS-pink1-RNAiKK30B VDRC 109614
w[*]; P{w[+mC]=UAS-Pink1.C}A BDSC 51648
w1118; ; UAS-Drp1-RNAiGD VDRC 44155
w[*]; P{w[+mC]=UAS-Drp1.D}3 BDSC 51647
w1118; UAS-Atg1-RNAiGD VDRC 16133
w1118; ; UAS-Atg1, 3xHA FlyORF F003538
w1118; ; UAS-ewg-RNAiGD VDRC 4560
w1118; UAS-CG7772-RNAiKK30B VDRC 100699
W; ; ato-Gal4-14a, UAS-CD8::GFP Hassan Lab W+A31
W; Sco / CyO, dtdYFP Hassan Lab W+ Bal II
W; ; Dr / TM3, Sb, dtdYFP Hassan Lab W+ Bal III
w1118; L / CyO; D / TM6C, Sb, Tb Hassan Lab Gb11
y[1] w[*] P{y[+t7.7] w[+mC]=UAS-myrGFP.QUAS-mtdTomato-3xHA}su(Hw)attP8; P{y[+t7.7] w[+mC]=trans-Tango}attP40 BDSC 77124
MCFO-1: pBPhsFlp2::PEST; ;10UAS-HA_V5_FLAG FlyLight, Janelia
w1118; UAS-Kir2.1 / CyO Hassan Lab
w1118; UAS-TNT-C / CyO Hassan Lab
w1118; ;UAS-dOrk1delta-NC(1) / TM3 Hassan Lab
w1118; ;UAS-dOrk1delta-C(1) / TM3 Hassan Lab

Wild animals	Not applicable
Reporting on sex	Not applicable
Field-collected samples	Not applicable
Ethics oversight	Wild-type rats were of the Sprague-Dawley strain Crl: CD (SD) and were bred by Janvier Labs (France) following the international genetic standard protocol (IGS). All procedures relating to the care and treatment of animals were performed following the guidelines of the European Directive 2010/63/EU and the French Decree n° 2013-118 concerning the protection of animals used for scientific purposes.

Note that full information on the approval of the study protocol must also be provided in the manuscript.

Clinical data

Policy information about clinical studies

All manuscripts should comply with the ICMJE guidelines for publication of clinical research and a completed CONSORT checklist must be included with all submissions.

Clinical trial registration	Not applicable
Study protocol	Not applicable
Data collection	Not applicable
Outcomes	Not applicable

Dual use research of concern

Policy information about dual use research of concern

Hazards

Could the accidental, deliberate or reckless misuse of agents or technologies generated in the work, or the application of information presented in the manuscript, pose a threat to:

No	Yes	
☒	☐	Public health
☒	☐	National security
☒	☐	Crops and/or livestock
☒	☐	Ecosystems
☒	☐	Any other significant area

Experiments of concern

Does the work involve any of these experiments of concern:

No	Yes	
☒	☐	Demonstrate how to render a vaccine ineffective
☒	☐	Confer resistance to therapeutically useful antibiotics or antiviral agents
☒	☐	Enhance the virulence of a pathogen or render a nonpathogen virulent
☒	☐	Increase transmissibility of a pathogen
☒	☐	Alter the host range of a pathogen
☒	☐	Enable evasion of diagnostic/detection modalities
☒	☐	Enable the weaponization of a biological agent or toxin
☒	☐	Any other potentially harmful combination of experiments and agents

Plants

Seed stocks	Not applicable
Novel plant genotypes	Not applicable
Authentication	Not applicable

ChIP-seq

Data deposition

☐ Confirm that both raw and final processed data have been deposited in a public database such as GEO.

☐ Confirm that you have deposited or provided access to graph files (e.g. BED files) for the called peaks.

Data access links<br>*May remain private before publication.*	No new ChIP seq data were generated
Files in database submission	Not applicable
Genome browser session<br>(e.g. UCSC)	Not applicable

Methodology

Replicates	Not applicable
Sequencing depth	Not applicable
Antibodies	Not applicable
Peak calling parameters	Not applicable
Data quality	Not applicable
Software	Not applicable

Flow Cytometry

Plots

Confirm that:

☐ The axis labels state the marker and fluorochrome used (e.g. CD4-FITC).

☐ The axis scales are clearly visible. Include numbers along axes only for bottom left plot of group (a 'group' is an analysis of identical markers).

☐ All plots are contour plots with outliers or pseudocolor plots.

☐ A numerical value for number of cells or percentage (with statistics) is provided.

Methodology

Sample preparation	Not applicable
Instrument	Not applicable
Software	Not applicable
Cell population abundance	Not applicable
Gating strategy	Not applicable

☐ Tick this box to confirm that a figure exemplifying the gating strategy is provided in the Supplementary Information.

Magnetic resonance imaging

Experimental design

Design type	No MRI imaging was performed
Design specifications	*Specify the number of blocks, trials or experimental units per session and/or subject, and specify the length of each trial or block (if trials are blocked) and interval between trials.*
Behavioral performance measures	*State number and/or type of variables recorded (e.g. correct button press, response time) and what statistics were used to establish that the subjects were performing the task as expected (e.g. mean, range, and/or standard deviation across subjects).*

Acquisition

Imaging type(s)	*Specify: functional, structural, diffusion, perfusion.*
Field strength	*Specify in Tesla*
Sequence & imaging parameters	*Specify the pulse sequence type (gradient echo, spin echo, etc.), imaging type (EPI, spiral, etc.), field of view, matrix size, slice thickness, orientation and TE/TR/flip angle.*
Area of acquisition	*State whether a whole brain scan was used OR define the area of acquisition, describing how the region was determined.*

Diffusion MRI ☐ Used ☐ Not used

Preprocessing

Preprocessing software	*Provide detail on software version and revision number and on specific parameters (model/functions, brain extraction, segmentation, smoothing kernel size, etc.).*
Normalization	*If data were normalized/standardized, describe the approach(es): specify linear or non-linear and define image types used for transformation OR indicate that data were not normalized and explain rationale for lack of normalization.*
Normalization template	*Describe the template used for normalization/transformation, specifying subject space or group standardized space (e.g. original Talairach, MNI305, ICBM152) OR indicate that the data were not normalized.*
Noise and artifact removal	*Describe your procedure(s) for artifact and structured noise removal, specifying motion parameters, tissue signals and physiological signals (heart rate, respiration).*
Volume censoring	*Define your software and/or method and criteria for volume censoring, and state the extent of such censoring.*

Statistical modeling & inference

Model type and settings — *Specify type (mass univariate, multivariate, RSA, predictive, etc.) and describe essential details of the model at the first and second levels (e.g. fixed, random or mixed effects; drift or auto-correlation).*

Effect(s) tested — *Define precise effect in terms of the task or stimulus conditions instead of psychological concepts and indicate whether ANOVA or factorial designs were used.*

Specify type of analysis: ☐ Whole brain ☐ ROI-based ☐ Both

Statistic type for inference (See Eklund et al. 2016) — *Specify voxel-wise or cluster-wise and report all relevant parameters for cluster-wise methods.*

Correction — *Describe the type of correction and how it is obtained for multiple comparisons (e.g. FWE, FDR, permutation or Monte Carlo).*

Models & analysis

n/a	Involved in the study
☐	☐ Functional and/or effective connectivity
☐	☐ Graph analysis
☐	☐ Multivariate modeling or predictive analysis

Functional and/or effective connectivity — *Report the measures of dependence used and the model details (e.g. Pearson correlation, partial correlation, mutual information).*

Graph analysis — *Report the dependent variable and connectivity measure, specifying weighted graph or binarized graph, subject- or group-level, and the global and/or node summaries used (e.g. clustering coefficient, efficiency, etc.).*

Multivariate modeling and predictive analysis — *Specify independent variables, features extraction and dimension reduction, model, training and evaluation metrics.*
