# Supplementary material for: Temporal transcriptional regulation of mitochondrial morphology primes activity-dependent circuit connectivity
Source: Nat Commun. 2025 Sep 1;16:8173. doi: 10.1038/s41467-025-62908-2 (PMC12402207; doi:10.1038/s41467-025-62908-2)
Supplement: Supplementary file 4 — Supplementary table [file 41467_2025_62908_MOESM4_ESM.pdf]

Peaks found

Category	Gene name Drosophila	Human homolog	Function	Number of peaks by gene
	CG7101	***TZAP***	Transcription factor	1
Mito transport	*milt*	*TRAK1/TRAK2*	Mitochondria transport	6
	Miro	*RHOT1/RHOT2*	Mitochondria transport	1
Mitochondria dynamic and architecture	***Drp1***	***DRP1***	Mitochondrial fision	1
	ewg	***NRF1***	Mitochondria biogenesis	2
	Marf	***MFN1/2***	Mitochondrial fusion	1
	Mitofilin	*IMMT/MIC60*	Cristae architecture	2
	cnc/Nrf2	*NFE2L1/NFE2L2/NFE2L3*	oxidative stress genes regulation	6
	Tom20	*MTOMM20*	Mitochondrial import	1
Autophagy and quality control	***park***	***PRKN***	Quality control	1
	Pink1	***PINK1***	Quality control	1
	ref(2)P	*SQSTM1*	Quality control	1
	Atg1	***ULK1***	Mitophagy	2
	Atg12	*ATG12*	Autophagy	3
	Atg5	*ATG5*	Autophagic vesicle formation	2
	Atg7	*ATG7*	Autophagy	2
mtDNA replication and maintenance	*bor*	*ATAD3A*	Component of nucleoids	2
	SCSα1	*SUCLG1*	Mitochondrial nucleotide synthesis	1
	mtDNA-helicase	*TWNK*	mtDNA replication	1
	mtSSB	*SSBP1*	mtDNA replication	1
	mtTFB1	*TFB1M*	Mitochondrial transcription	1
	mtTFB2	*TFB2M*	Mitochondrial transcription	2

Category	Gene name Drosophila	Human homolog	Function	Number of peaks by gene
Mitochondria translation	*mRpL1*	*MRPL1*	Mitoribosomes subunits	1
	mRpL10	*MRPL10*		1
	mRpL11	*MRPL11*		1
	mRpL13	*MRPL13*		1
	mRpL16	*MRPL16*		1
	mRpL17	*MRPL17*		1
	mRpL18	*MRPL18*		2
	mRpL19	*MRPL19*		1
	mRpL2	*MRPL2*		1
	mRpL20	*MRPL20*		1
	mRpL21	*MRPL21*		1
	mRpL23	*MRPL23*		2
	mRpL24	*MRPL24*		1
	mRpL27	*MRPL27*		3
	mRpL28	*MRPL28*		1
	mRpL32	*MRPL32*		1
	mRpL33	*MRPL33*		1
	mRpL34	*MRPL34*		1
	mRpL35	*MRPL35*		1
	mRpL37	*MRPL37*		1
	mRpL39	*MRPL39*		1
	mRpL4	*MRPL4*		1
	mRpL41	*MRPL41*		1
	mRpL43	*MRPL43*		1
	mRpL44	*MRPL44*		1
	mRpL47	*MRPL47*		1
	mRpL48	*MRPL48*		1
	mRpL50	*MRPL50*		1
	mRpL51	*MRPL51*		1
	mRpL52	*MRPL52*		2
	mRpL53	*MRPL53*		1
	mRpL54	*MRPL54*		1
	mRpL55	*MRPL55*		2
	mRpL9	*MRPL9*		1

Category	Gene name Drosophila	Human homolog	Function	Number of peaks by gene
Complex I	*ND-18*	*NDUFS4*	Supernumerary subunit	1
	ND-20	*NDUFS7*	Core subunit	1
	ND-23	*NDUFS8*	Core subunit	1
	ND-39	*NDUFA9*	Supernumerary subunit	2
	ND-42	*NDUFA10*	Supernumerary subunit	1
	ND-51	*NDUFV1*	Core subunit	1
	CIA30	*NDUFAF1*	Assembly factor	2
Complex II	*SdhA*	*SDHA*	Subunit	2
	Sdhaf3	*SDHAF3*	Assembly factor	1
	SdhB	*SDHB*	Subunit	1
	SdhC	*SDHC*	Subunit	2
	Sirup	*SDHAF4*	Assembly factor	1
Complex III	*Ttc19*	*TTC19*	Assembly factor	1
	Ox	*UQCR10*	Supernumerary subunit	2
Complex IV	*Ccdc56*	*COA3*	Assembly factor	1
	COX5A	*COX5A*	Citochrome C oxidase subunit	1
	COX5B	*COX5B*	Suplementary subunit	1
	COX7A	*COX7A1*	Suplementary subunit	1
Complex V	*ATPsynB*	*ATP5PB*	Core subunit	1
	ATPsynC	*ATP5MC1/ ATP5MC2/ATP5MC3*	Core subunit	3
	ATPsynD	*ATP5PD*	Core subunit	3
CoQ	*qless*	*PDSS1*	CoQ biosynthesis	2

No peaks

Gene name Drosophila	Human homolog	Function	Number of peaks by gene	Category
srl	***PCG1a/PPARGC1A***	Transcriptional coactivator	0	
opa1	***OPA1***	Mitochondrial fusion	0	Mitochondrial dynamics
Mic26-27	*MIC26-27*	Cristae architecture	0	
Slc25A46a	***SLC25A46***	Mitochondria dynamics	0	
Fis1	***FIS1***	Mitochondria fission	0	
PolG1/tam	*POLG*	mtDNA replication	0	mtDNA replication and transcription
TFAM	*TFAM*	mtDNA transcription and replication	0	
mTTF	*MTERF1*	mtDNA transcription	0	
mRpL15	*MRPL15*	Mitoribosome large subunit	0	Mitochondrial translation
tko	*MRPS12*	Mitoribosome small subunit	0	
mRpL41	*MRPL41*	Mitoribosome subunit	0	
mt:ND2	*MT_ND2*	Core subunit	0	Complex I
sloth1	*SMIM4/UQCC5*	Assembly factor	0	Complex III
sloth2	*Brawnin/UQCC6*	Assembly factor	0	
Bcs1	*BCS1L*	Assembly factor	0	
mt:Coi	*MT-CO1*	Core subunit	0	Complex IV
Coa7	*COA7*	Assembly factor	0	
Coa8	*COA8*	Assembly factor	0	
Surf1	*SURF1*	Assembly factor	0	
Scox	*SCO1/SCO2*	Assembly factor	0	

Supplementary Table 1. Number of normalized peaks for CG7101-GFP ChipSeq data sets from whole female fly (ModENCODE project). We were able to detect putative binding of CG7101/Mirana protein to numerous genes involved in mitochondria function and homeostasis.
